# Are Local Filters Blind to Provenance? Ant Seed Predation Suppresses Exotic Plants More than Natives

**DOI:** 10.1371/journal.pone.0103824

**Published:** 2014-08-06

**Authors:** Dean E. Pearson, Nadia S. Icasatti, Jose L. Hierro, Benjamin J. Bird

**Affiliations:** 1 Rocky Mountain Research Station, United States Department of Agriculture, Forest Service, Missoula, Montana, United States of America; 2 Division of Biological Sciences, University of Montana, Missoula, Montana, United States of America; 3 Instituto de Ciencias de la Tierra y Ambientales de La Pampa (Consejo Nacional de Investigaciones Científicas y Técnicas-Universidad Nacional de La Pampa) and Facultad de Ciencias Exactas y Naturales, Universidad Nacional de La Pampa, Santa Rosa, La Pampa, Argentina; 4 Rocky Mountain Research Station, United States Department of Agriculture, Forest Service, Fort Collins, Colorado, United States of America; Key Laboratory of Tropical Forest Ecology, Xishuangbanna Tropical Botanical Garden, Chinese Academy of Sciences, China

## Abstract

The question of whether species’ origins influence invasion outcomes has been a point of substantial debate in invasion ecology. Theoretically, colonization outcomes can be predicted based on how species’ traits interact with community filters, a process presumably blind to species’ origins. Yet, exotic plant introductions commonly result in monospecific plant densities not commonly seen in native assemblages, suggesting that exotic species may respond to community filters differently than natives. Here, we tested whether exotic and native species differed in their responses to a local community filter by examining how ant seed predation affected recruitment of eighteen native and exotic plant species in central Argentina. Ant seed predation proved to be an important local filter that strongly suppressed plant recruitment, but ants suppressed exotic recruitment far more than natives (89% of exotic species vs. 22% of natives). Seed size predicted ant impacts on recruitment independent of origins, with ant preference for smaller seeds resulting in smaller seeded plant species being heavily suppressed. The disproportionate effects of provenance arose because exotics had generally smaller seeds than natives. Exotics also exhibited greater emergence and earlier peak emergence than natives in the absence of ants. However, when ants had access to seeds, these potential advantages of exotics were negated due to the filtering bias against exotics. The differences in traits we observed between exotics and natives suggest that higher-order introduction filters or regional processes preselected for certain exotic traits that then interacted with the local seed predation filter. Our results suggest that the interactions between local filters and species traits can predict invasion outcomes, but understanding the role of provenance will require quantifying filtering processes at multiple hierarchical scales and evaluating interactions between filters.

## Introduction

Efforts to understand biological invasions have increased exponentially in recent decades, yet advances in understanding and predicting invasion outcomes remain elusive [1]. In fact, debate continues as to whether the processes affecting exotic species invasions differ at all from those affecting native colonization [2], [3]. Theoretically, communities are assembled by biotic and abiotic processes acting from regional to local scales to filter individual species based on their functional traits [4]; a process presumably blind to species origins. The question of whether provenance plays an important role in invasion could be tested by manipulating filters and quantifying native and exotic species responses. Moreover, if local filters are examined, such an approach could also elucidate the role of community context in invasion outcomes. Currently, community context is not well integrated into invasion research, a situation hindering progress in this field. For example, the extensive efforts made to predict invader success by evaluating invader traits independent of community context have met with limited success [5], [6]. In contrast, accumulating studies suggest that invasion outcomes are best understood in the context of community-specific processes [7]–[9].

Historically, competition has been emphasized as the central local process structuring plant communities [10]. However, seed predation can also profoundly affect plant community structure [11]. A rapidly growing body of work indicates that rodent seed predation is a powerful local filter to plant recruitment that can also influence plant invasions [12]–[18]. Selective foraging by rodents, particularly with regard to seed size, can suppress certain exotic species, causing long-term and wide-spread population reductions [14], [17], but it can also prove advantageous for exotics that evade those seed predation pressures that inhibit their potential competitors [13]. Ants are also important seed predators that can influence plant community structure via selective seed predation [19]–[23]. However, most work on ant-seed interactions has focused on seed dispersal or examined spatial or temporal aspects of seed removal [24], while relatively few studies have experimentally quantified the effects of ant seed predation on plant recruitment [19]–[23]. Moreover, the relative effects of ant seed predation on native versus exotic plant recruitment are unknown. In central Argentina, we conducted seed offering and seed addition experiments to examine the importance of ant seed predation as a local filter to plant recruitment and to quantify its relative effects on recruitment of nine native and nine exotic plant species.

## Methods

### Ethics Statement

The research permit was issued by the Subsecretaría de Ecología, Gobierno de La Pampa, Av. Luro 700, (6300) Santa Rosa, La Pampa, Argentina, and signed by Lic. Fabián Tittarelli. This research did not involve any vertebrate subjects or any endangered or protected species.

### Study system

We conducted our study in Parque Luro Provincial Reserve, a 7,500-ha park 30 km south of Santa Rosa, in La Pampa Province, central Argentina (36° 54′ 33″ S, 64° 15′ 38″ W). The park lies within the Caldenal vegetation type, a forest/savanna habitat dominated by the tree *Prosopis caldenia.* The park contains largely intact native plant communities, but has extensive disturbed areas that are invaded by exotics. There is no domestic grazing in the park, but wild herbivores are present. We focused on open grassland-savanna habitat where understory vegetation was dominated primarily by the native grasses *Nassella tenuissima* and *Piptochaetium naposteanse*, with *Solanum* spp. and *Baccharis* spp. among the more common native forbs. All experiments described were replicated at 10 sites scattered across the park (1–10 km spacing).

We examined nine exotic herbaceous plant species (Table 1) that range from highly abundant in the study area (e.g., *Diplotaxis tenuifolia*, *Centaurea solstitialis*, *Chenopodium album*) to sporadic or uncommon (e.g., *Hypochaeris radicata*, *Taraxacum officinale, Rumex crispus*). The nine species represent 15% of 62 exotics identified within the region in recent large-scale surveys (DE Pearson, JL Hierro, D Villarreal, unpubl data). We also selected nine widespread native herbaceous plant species that similarly vary in local abundance (Table 1). The only ant species identified at our experimental sites was *Pheidole bergi*, a social harvester ant, which is insectivorous and granivorous and widespread across Argentina and surrounding countries [25]. Although other ant species are presumably present in the area [26], this species appears to dominate.

**Table 1:** A study species list with information on origin (N = native, E = exotic), mean seed mass (g), general life history characteristics (F = forb, G = grass, A = annual, B = biennial), and whether seeds have elaiosomes (fatty bodies attached to the seed that have evolved for ant seed dispersal).

Species	Family	Life history	Seed mass	Elaiosome	Origin
*Bromus catharticus*	Poaceae	G, A	0.00555	No	N
*Carduus nutans*	Asteraceae	F, A	0.00367	Yes	E
*Cenchrus incertus*	Poaceae	G, A	0.00642	No	N
*Centaurea solstitialis* (with pappus)	Asteraceae	F, A or B	0.00198	No^a^	E
*Centaurea solstitialis* (without pappus)	Asteraceae	F, A or B	0.0014	No	E
*Chenopodium album*	Chenopodiaceae	F, A	0.0005	No	E
*Daucus pusillus*	Apiaceae	F, A	0.0016	No	N
*Diplotaxis tenuifolia*	Brassicaceae	F, P	0.00015	No	E
*Gaillardia megapotamica*	Asteraceae	F, P	0.00215	No	N
*Hordeum euclaston*	Poaceae	G, A	0.00326	No	N
*Hordeum stenostachys*	Poaceae	G, P	0.00453	No	N
*Hypochaeris radicata*	Asteraceae	F, P	0.0006	No	E
*Rumex crispus*	Poligonaceae	F, P	0.0014	No	E
*Salsola kali*	Chenopodiaceae	F, A	0.00155	No	E
*Solanum elaeagnifolium*	Solanaceae	F, P	0.00654	No	N
*Taraxacum officinale*	Asteraceae	F, P	0.00032	No	E
*Thelesperma megapotamicum*	Asteraceae	F, P	0.00237	No	N
*Tragopogon dubius*	Asteraceae	F, A or B	0.00902	No	E
*Verbesina encelioides*	Asteraceae	F, A	0.00293	No	N

a Pemberton and Irving (1990) concluded that *C. solstitialis* seeds lack elaiosomes, but the pappus-bearing seeds of this plant have structures similar to those described as very poorly developed elaiosomes in other species in this genus.

Ants can destroy seeds. However, they can also facilitate plant recruitment through seed dispersal and ecosystem engineering [24]. Hence, determining the effects of ants on plant recruitment requires understanding not only how ant seed preferences influence seed removal, but also how seed removal relates to seed fate and ultimately plant establishment. We evaluated seed preference [27] and its effects on seed fate using two seed offering experiments. We quantified ant effects on plant recruitment using a seed addition experiment.

### Seed Preference and Seed Fate

**Figure 1:**
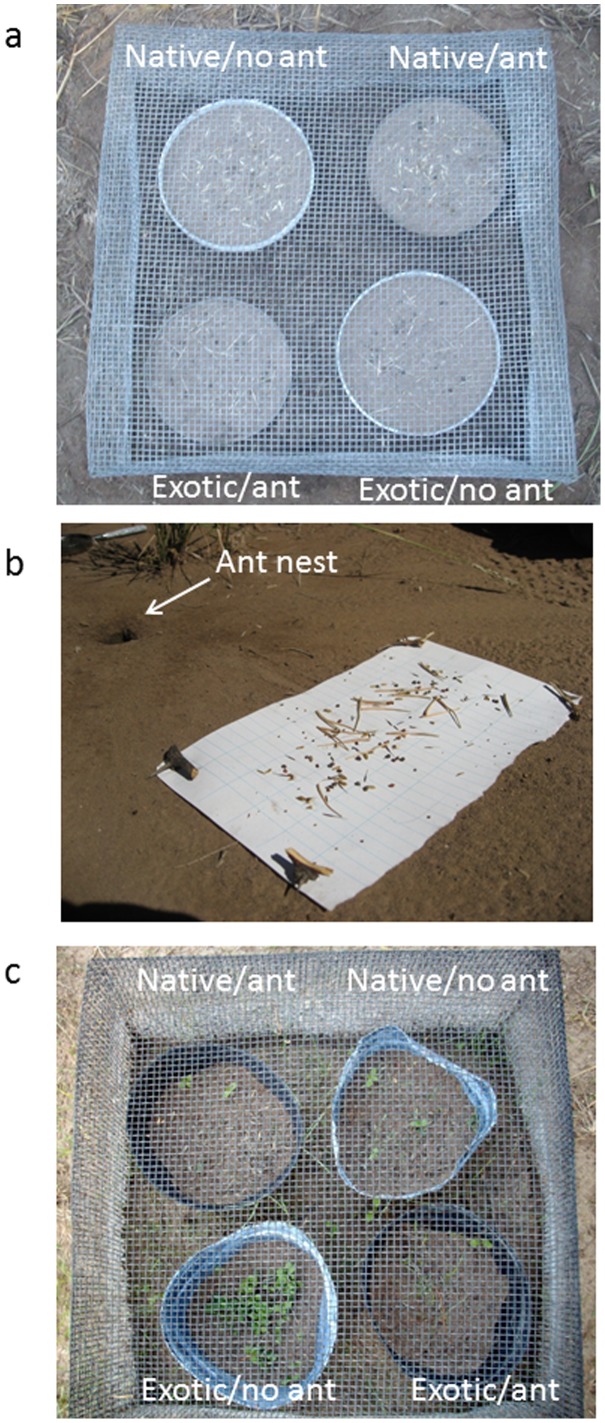
Photographs illustrating the experimental design. a) seed preference experiment, b) seed fate experiment, and c) seedling recruitment experiment.

In our first seed offering experiment, we examined ant seed preference using a multiple choice preference design [28] by setting seeds out in Petri dishes for 5 day-periods in February of 2010 and 2011 (Fig. 1a). Seed offering stations were located randomly and independent of ant nests at each of the 10 study sites. At each site, four Petri dishes (15 cm dia.×1.5 cm tall) were assigned to one of four treatments based on species origins (exotic or native, with each dish containing all exotic or all native species in a multiple choice design) and ant access (access or no access) in a full factorial design. Ant access was precluded in preference trials as a control for evaluating the effects of environmental factors such as wind or rain in displacing seeds from dishes. In each set of offerings, Petri dishes were placed 10 cm apart within a hardware cloth cage (40 cm×40 cm wide and ×20 cm high; mesh size 0.5 cm×0.5 cm) to exclude vertebrates. In treatments excluding ant access, dishes were placed on nails four cm above the ground and the outer surfaces of the dishes and nails were painted with fluon, a slippery compound that ants cannot climb [29]. Ant access dishes were placed directly on the ground. Our observations indicated that the ants could not breach the fluon barrier and seed offering results confirmed these observations (see Results). Petri dishes were filled three quarters full with soil and twenty seeds of each species were placed on the soil surface. Because *C. solstitialis* produces two types of seeds (with and without a pappus) which differ in seed mass (Table 1), we included both types of seeds from this plant as though they were from different species. Hence, in the exotic seed offerings there were 10 seed types and 200 total seeds and in the native seed offerings there were 9 seed types and 180 total seeds.

Our second seed offering experiment focused on seed fates, but also quantified seed preferences closer to ant nests. In this experiment, we set out seeds on field notebook paper (10 cm×25 cm) that was pinned to the ground 15 cm from a randomly selected ant nest at each study site to visually quantify seed removal and seed fates (Fig. 1b). We executed two versions of this experiment, both in February 2010. In the first version, we offered seeds of first all nine exotic and then all nine native species in two separate trials at each site. In each trial, we placed 20 seeds of each species onto the paper and recorded seed removal/fate over a 60-min period by quantifying the number of seeds per species 1) removed and taken into the nest, 2) removed and dropped before reaching the nest, or 3) not removed. As in the previous experiment, the two *C. solstitialis* seed types were included as though separate species. In the second version of this experiment, we set out seeds of the four most preferred exotic and native species (determined from the first version of this experiment) simultaneously using the same approach in order to more directly compare preference for the different seed types (i.e., eight total species, four exotic and four native, 20 seeds per species offered once at each site).

Since seeds taken into the nest can later be deposited above ground in refuse piles or “basuras” near the nest entrance where they can germinate or be further dispersed, we also evaluated the number and viability of seeds that resurfaced from the nests. To do this, prior to setting seeds out for the seed offering experiments above, we swept the basuras away from the nest openings until only bare ground remained. We then revisited the nests four days after the seed offering experiment to collect the new basuras. Basura contents were taken to the lab where all target species’ seeds were extracted, identified, and designated as either destroyed (endosperm damaged or only seed coats remaining) or whole and potentially viable. Potentially viable seeds were tested for germination by placing them on filter paper floating on water in Perti dishes for 10 days and checking for germination. Seeds that did not germinate were tested for viability using tetralozium [30]. These results were used to reassign seed fates for seeds that emerged from the nest as either incapacitated (destroyed or emerged nonviable) or secondarily dispersed. Seeds that did not emerge from the nest in four days were presumed incapacitated (unlikely to emerge from the nest still viable). In 30 hours of nest observations, we did not observe ants discarding materials in locations other than the basuras. All seeds for all experiments were collected from local populations in the same season they were offered. Seed mass for each species was determined by weighing 50 seeds and calculating average seed mass.

### Seedling Recruitment

To evaluate the effects of ant seed predation on plant recruitment, we conducted seed addition experiments at three locations (30–60 m apart) within each of the 10 study sites in 2011. At each location, we placed seeds in four plastic greenhouse pots (16 cm dia×15 cm tall) that were assigned to one of four treatments based on species origins (exotic or native) and ant access (access or no access) in a full factorial design (Fig. 1c). Pots were spaced 5 cm apart, buried 9 cm into the soil, filled to 9 cm depth with soil from the site, and covered with hardware cloth cages (same dimensions as above) anchored to the soil surface to prevent vertebrate access. Each pot received 20 seeds from each of the nine species of exotics or natives. Only pappus-bearing seeds of *C. solstitialis* were used in this experiment. Pots assigned to ant access were perforated with eight 1-cm dia holes at the soil surface. Pots assigned to no ant access remained unperforated and were painted with fluon along interior and exterior exposed surfaces. Experiments were initiated in March 2011 and seedlings were counted 15, 30, 60, and 90 days following the first rain of ≥20 cm, which generally initiates germination in this system.

### Analyses

All analyses were conducted using R [31]. We used the same analytical framework to examine 1) seed preference of ants, as measured by the relative number of seeds removed per species in each of the seed offering experiments, and 2) the effect of ant access on seedling emergence during the second sampling period, which approximated peak emergence for most species. Since multiple species of seeds were set out together in each experiment, responses were not independent among species. To address this issue, we used the multivariate Hotelling’s T^2^ test [32] to test for differences between species and species’ origins in the 60-min seed offerings or between ant access and no access treatments in the five-day seed offering and seedling emergence experiments. When applying the Hotelling’s T^2^ test, the treatment and control trials were considered as two multivariate populations with separate covariance structures [32]. For these tests, we used Yao’s [33] degrees-of-freedom approximation [32]. For the five-day seed offering experiments, seed losses due to environmental factors were addressed by adjusting for losses from ant access dishes using estimates from the paired no-ant access control dishes before applying Hotelling’s T^2^ test [32]. In the 60-min seed offering experiments, all seed fates were observed, so adjusting for environmental losses was unnecessary.

As assurance against violations of multivariate normality assumptions, we followed up the Hotelling’s T^2^ tests with nonparametric Friedman’s rank sum tests [34], which do not depend on multivariate assumptions but do not account for the multivariate nature of the responses. Since in all cases the two methods produced the same qualitative outcomes and rejected the null hypotheses, we used results from the Friedman’s tests followed up with post-hoc tests evaluating difference between species with a Bonferroni correction to control for the lack of independence among tests. Results from the Friedman’s tests for individual species responses should not be interpreted as independent of the other species in the experiment, i.e., the results are presumed conditioned by the composition of the seeds set out. Combining these methods provided a complimentary means of addressing these data complexities. Moreover, the relative rankings from the Friedman’s tests provided indices for further analyses. For the five-day seed offering trials, the Friedman’s test generated sums of the rankings of relative seed removal (adjusted for environmental effects) that served as an index of preference for the seed offering experiments with higher values indicating greater preference. These are computed in the Freidman’s test as sums of the ranked values for the number of removed seeds after adjusting the counts for environmental loss based on the controls. For the seedling recruitment experiments, the Friedman’s rankings (which are derived from the relative ranking of the difference in recruitment between the paired ant access and no access cages) served as an index of ant impacts on seedling recruitment, with higher values indicating greater impact on recruitment (i.e., ant access results in lower recruitment). Both approaches generate relative rankings across the species that are bounded by zero at the bottom with upper limits restricted by the amount of data (i.e., the number of species considered and/or the number of replicate cages).

The above tests as applied to seedling emergence demonstrate differences in ant impacts on emergence across species. However, we also wished to more explicitly evaluate the effects of ant access on the absolute number of seedlings recruiting across species. To do this, we applied a MANOVA with ant exclosure treatment and site as fixed effects and number of seedlings (log [seedling number +0.5]) as the dependent variable, using mean seedling recruitment across sampling periods. Residual diagnostics indicated no violations of MANOVA assumptions.

We tested for differences in seed fates between exotics and natives by comparing the proportion of removed seeds that were incapacitated using a GLM fitted to a beta distribution after applying a shrinkage transformation to address zeros [35]. We evaluated the relationship between log transformed seed mass and the preference indices from the five-day seed offering experiments using linear regression. We examined models with and without *C. nutans* because its seeds have a well-developed elaiosome (Table 1), a fatty appendage that is adapted for ant seed dispersal [36]. We also used linear regression to examine the relationship between preference and ant impacts on plant recruitment using the seed preference index from the five-day offerings and the index for impacts on plant recruitment. We compared mean seed masses between exotic and native species using ANOVA with seed origins as the independent variable. To examine whether the seed masses of the nine exotics used in our experiments were unusual, we similarly compared seed masses of our nine exotics with those of 16 other exotic herbaceous species from this system for which we had seed mass data. Seed masses were log transformed in both of these analyses to meet ANOVA assumptions (statistics are back-transformed least squares means and SEs). Finally, we evaluated the effect of seed mass versus seed origins on ant seed preference using ANOVA with log(seedmass) and seed origins treated as fixed factors. For this analysis, we excluded *C. nutans* because its elaiosome over-rode the effect of seed mass on ant preference.

We examined seedling emergence and timing of emergence between exotics and natives in the absence of ants to evaluate potentially different responses to abiotic conditions. For these analyses, we compared the average number of seedlings emerging (averaged across sampling dates) by plant origins using ANOVA and we compared peak emergence dates (15, 30, 60, 90 days) between exotics and natives using a Mann-Whitney test because normality assumptions were questionable. To determine whether seed mass was related to total emergence and timing of emergence, we examined the relationship between seed mass and average emergence (defined above) using ANOVA, but we used Spearman’s correlation test to examine the relationship between seed mass and peak timing of emergence as the data were non-normal.

## Results

**Figure 2:**
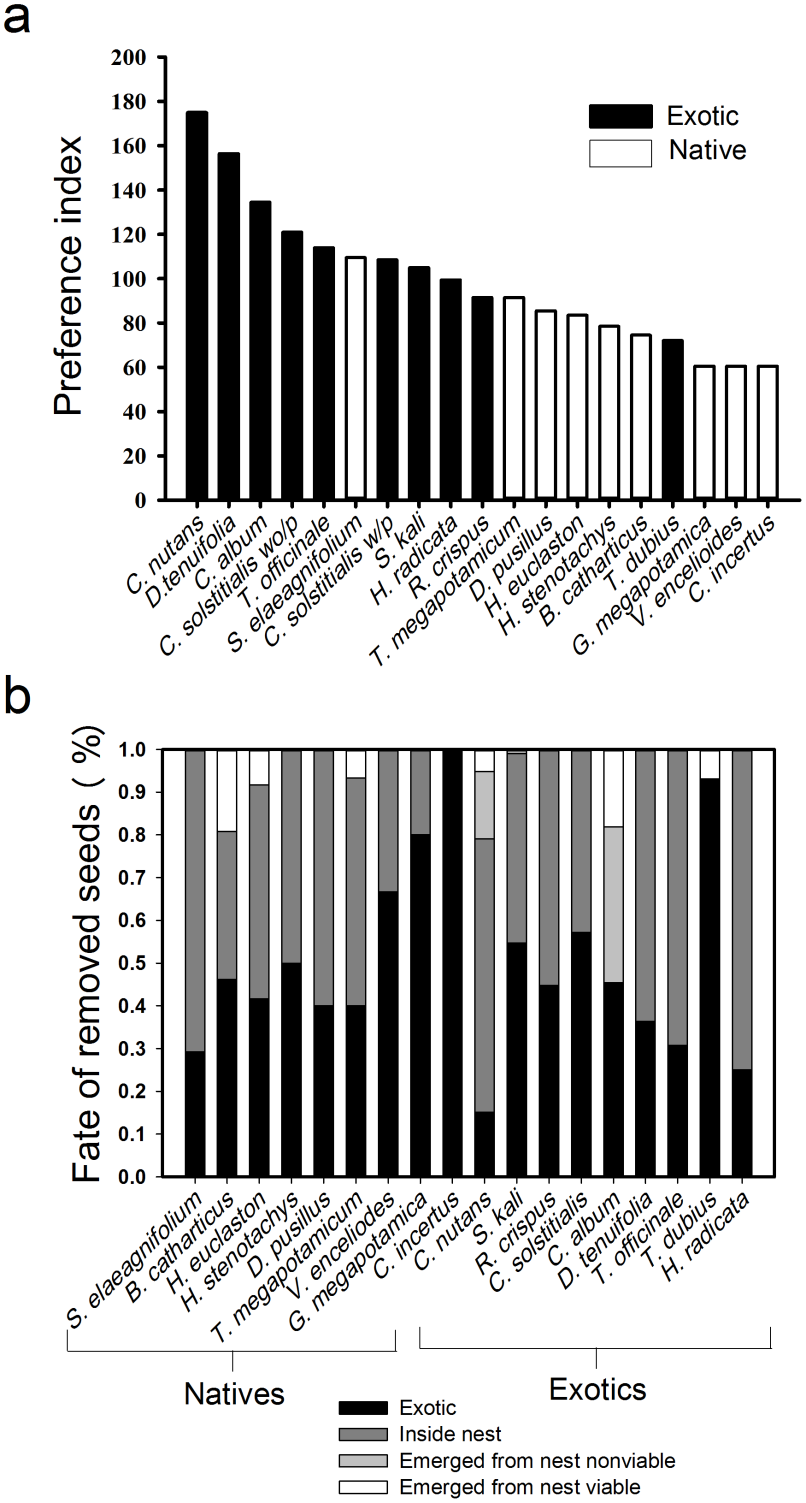
Ant seed preferences and their effects on seed fates. a) Preference of ants for native and exotic seeds based on seed removal from experimental seed depots set out over five days. Species that share letters above bars were not significantly different (Friedman’s test, *α* = 0.05). b) Fates of native and exotic seeds removed by ants during 60 min observations of experimental depots placed near nests.

**Figure 3:**
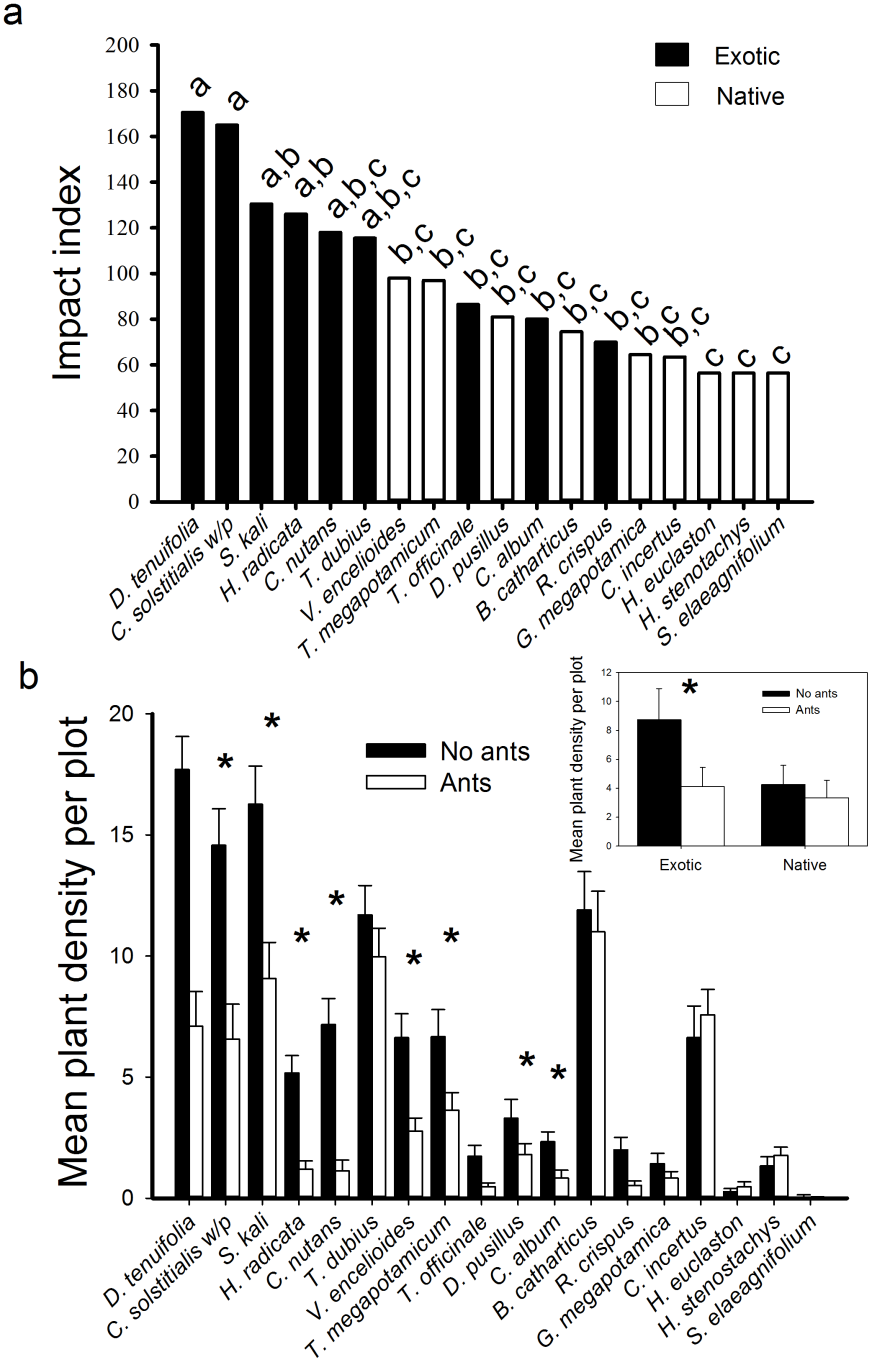
Ant impacts on plant recruitment. a) Index of ant impacts on number of seedlings recruiting based on seed addition experiments that allowed or precluded ant access to native and exotic seeds for the second sampling period, which approximated peak emergence for most species. Different letters above bars indicate significant difference among species (Friedman’s test, *α* = 0.05). b) Establishment of plants (mean ± SE) by the end of the growing season in plots exposed to or protected from ant seed predation. Asterisks indicate significant differences based on MANOVA tests for individual species (*α* = 0.05).

Ants exhibited strong and consistent seed preferences as determined by the proportions of seeds removed across all test species. Seed preferences determined from the five-day and the 60-min seed offering experiments (both versions) produced similar preference rankings across species, so we present preference results only for the five-day experiment. Seed preference rankings differed significantly among species (Friedman’s test χ^2^_18_ = 66.41, *P*<0.01), with exotics ranked as 9 of the 10 most preferred species (Fig. 2a). Overall, the proportion of seeds removed was significantly higher for exotic than for native species (Friedman’s test χ^2^_1_ = 6.4, *P* = 0.01). Seed fates determined from the 60-min seed offerings at ant nests indicated that 414 of 684 or 61% of ant-removed seeds were taken into the nests (Fig. 2b). The proportion of ant-removed seeds that were incapacitated (entered nests but did not emerge viable) did not differ between natives and exotics (χ^2^_1_ = 1.27, *P* = 0.26). Of the seeds we observed entering the nests; only 14% emerged from the nests and entered the basuras within four days. Thirty-eight percent of these emerging seeds or 5% of the total seeds that had entered the nests emerged still viable, suggesting that seed entry into the nests was largely fatal. The remaining 39% of removed seeds were dropped as ants returned to the nest, but we observed that many dropped seeds are later picked up by other colony members and transported to the nest. Seed preference predicted ant impacts on seedling recruitment (r^2^ = 0.26, *F*_1,16_ = 5.54, *P*<0.03). Ant impacts on seedling emergence varied across species (Friedman’s test χ^2^_17_ = 51.15, *P*<0.01), with seven of the ten most impacted species being exotics (Fig. 3a). Ant access to seeds substantially reduced the number of seedlings recruiting (Overall MANOVA; *F*_18,30_ = 5.2, *P*<0.001), with eight exotics and two natives showing significant reductions in recruitment (individual species tests; α = 0.05; Fig. 3b).

**Figure 4:**
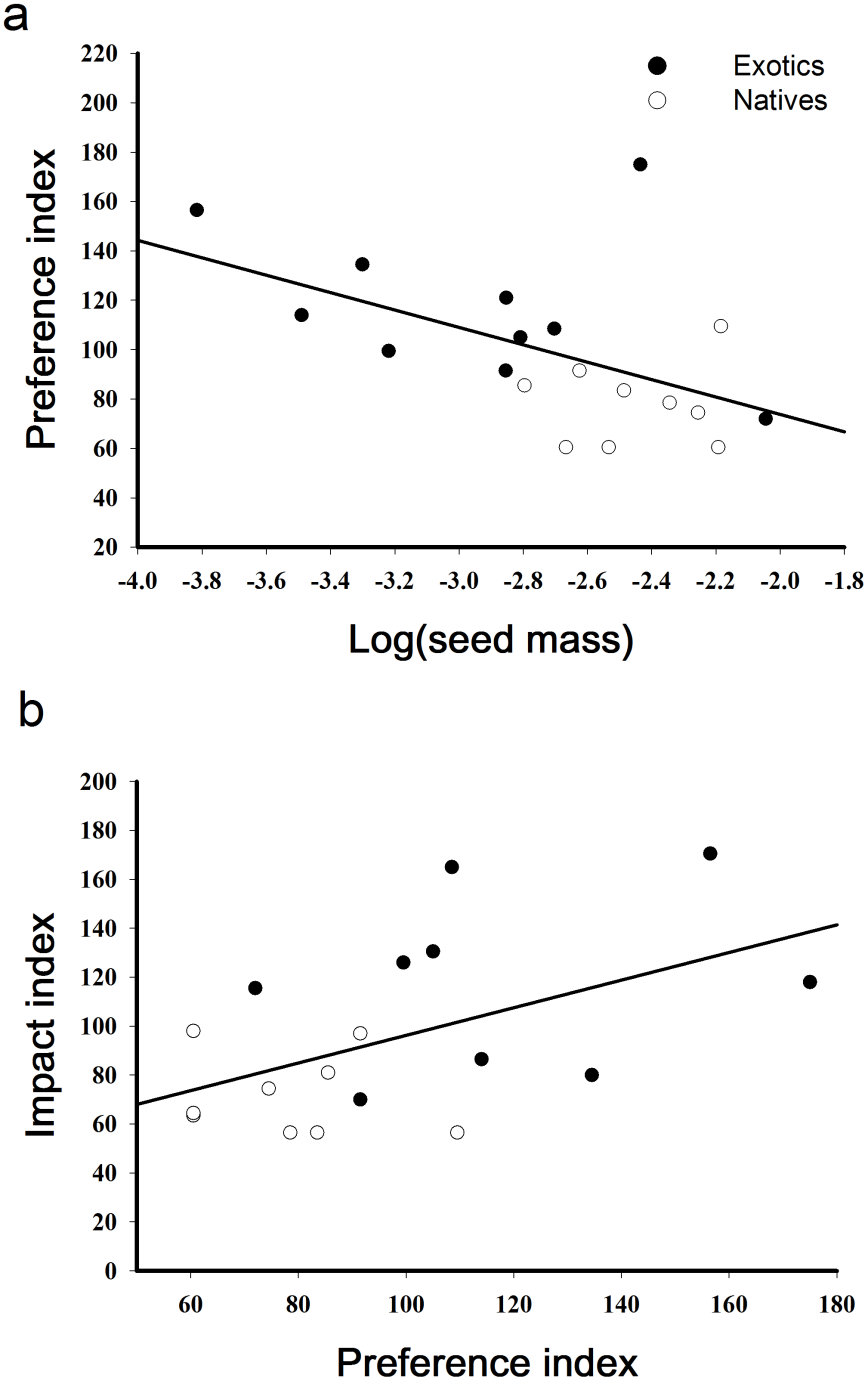
Correlations between seed mass and ant preference and ant preference and ant impacts on plant recruitment. a) Relationship between log (seed mass in grams) and ant preference for seeds of nine native and nine exotic plant species (the exotic *C. solstitialis* is represented by both pappus- and nonpappus-bearing seeds). The outlier is *C. nutans* which has a large well-develop elaiosome (see Discussion). b) Relationship between the seed preference index and the index for ant impacts on seedling recruitment for the same native and exotic species (*C. solstitialis* is represented by only its pappus-bearing seed).

Ant preference was strongly predicted by seed mass, with ants preferring smaller seeds (Fig. 4a; r^2^ = 0.28, *F*_1,17_ = 6.45, *P* = 0.02). The fit of this model doubled when the elaiosome-bearing *C. nutans* was removed from the data (r^2^ = 0.57, *F*_1,16_ = 21.31, *P*<0.01). Seed masses of exotics (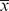 = 0.0011±0.003) were significantly smaller than those of natives (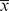 = 0.0035±0.0011; *F*_1,17_ = 7.12, *P* = 0.02). Seed masses of the nine exotics used in this study (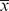 = 0.0011±0.0004) did not differ from seed masses of 16 other exotic plants in this system (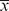 = 0.0022±0.0006; *F*_1, 24_ = 1.91, *P* = 0.18). Despite the general differences between exotics and natives in seed mass, seed mass (*F*_1, 15_ = 8.67, *P* = 0.01) determined ant seed preferences independent of plant origins (*F*_1, 15_ = 2.31, *P* = 0.15). In the absence of ants and vertebrate consumers, exotics exhibited earlier peak emergence (Mann-Whitney, *W* = 14, *P*<0.01; data not shown) and tended toward higher average emergence (*F*_1,16_ = 4.05, *P* = 0.06) than did the natives (Fig. 3b inset). Seed mass was not correlated with the average number of seedlings emerging (*F*_1,16_ = 0.001, *P* = 0.97) or the peak time of emergence (*S* = 756.71, *P* = 0.38).

## Discussion

In the Caldenal of central Argentina, we found that ant seed predation was a powerful local filter to plant recruitment. Ant preferences for smaller seed sizes resulted in substantially reduced recruitment of smaller-seeded species. Interestingly, this size-dependent seed selection resulted in far greater impacts on exotic plant recruitment - an outcome driven by the fact that seeds of exotics were smaller than those of natives in our system. Exotics also differed in that they exhibited greater emergence and earlier peak emergence than natives in the absence of seed predation. Remarkably, when the seed predation filter was in place it largely negated the differences in overall seedling emergence and timing between the groups due to the bias against exotics. The fact that exotics differed from natives in seed size, timing of emergence, and overall emergence success in the absence of seed predation suggests that higher-order filters such as introduction pathways or regional abiotic conditions may have preselected for certain exotic traits. These results suggest that the local seed predation filter acted on seed traits independent of plant origins, but that higher-order filters may have preselected for exotic traits that then interacted with the local filter to generate a provenance bias.

While ant seed dispersal has been studied extensively [24], [37], the effects of ant seed predation on plant recruitment is not well understood. One reason for this is that determining seed fates is logistically challenging, and without knowing seed fates it is unclear whether ants are acting as dispersers or consumers. We conducted experiments designed to quantify the fates of seeds removed by ants across our study species, including assessing viability of seeds that were taken into the nest and later returned to the soil surface. We found that 61% of seeds that were removed in 60-min trials were taken directly into the nest. Of these, only five percent emerged viable within four days. Hence, most seeds taken into the nests were likely incapacitated (i.e., consumed, buried too deep for emergence, or destroyed by pathogens). Some of the seeds which were removed from seed depots and dropped before reaching the nest likely experience secondary dispersal. However, many of the seeds we observed arriving at the nest during the 60-min trials had been dropped by the initial carrier and rediscovered by other ants that ultimately transported them to the nest. We expect that this process results in most of the dropped seeds ultimately entering the nests over more natural time frames. In this system, *P. bergi* foraging likely results in some seed dispersal, but the great majority of ant-collected seeds appear to be removed from the seed pool.

Our seed fate results provide valuable insights regarding how species introductions and the resultant shuffling of evolutionary histories and ecological contexts can influence invasion outcomes, particularly in the context of coevolved mutualisms. The exotic *C. nutans* has a well-developed elaiosome, a fatty appendage evolved in some seeds as a food reward for ants in exchange for seed dispersal, myrmecochory [24]. Not surprisingly then, *C. nutans* was the most preferred species in our study (Fig. 2a) despite having a relatively large seed size – a trait associated with avoidance in our ants. Our observations of ants consistently grabbing this species by the elaiosome substantiated the elaiosome’s role in influencing the ant’s preference for this seed. Yet, this species was the most negatively impacted by ant foraging (Fig. 3b). Ants commonly removed nearly all *C. nutans* seeds from the seed depots in ≤60 min, and of those that emerged from the nests only 25% or 6% of all removed seeds entering the nests were viable. *Pheidole* spp. can have largely destructive effects on elaiosome-bearing seeds in other systems as well [37].

Elaiosomes are most advantageous for myrmecochory when the ants involved are primarily carnivorous because carnivorous ants consume the fatty bodies, which emulate animal lipids, and dispose of the endosperm unharmed [36]. We show that in the wrong context elaiosomes can actually reduce plant fitness. Moreover, in reviewing the literature for herbaceous and woody plants in our system, we found evidence that elaiosomes were 24-times more common in introduced (14 of 157 species or 9%) versus native plants (1 of 258 species or 0.4%) (N. Icasatti unpublished data). Elaiosomes are generally rare among South American plants, in contrast to the Europe/Anatolia region, which is a hotspot for elaiosome evolution [38] and the region of origins for *C. nutans* [39]. These results demonstrate how traits evolutionarily adapted to one system can be maladaptive in the wrong ecological context. They also demonstrate the importance of determining seed fates to fully understand ant-plant interactions. Many of the studies that have concluded that elaiosomes benefit introduced plants via myrmecochory (including *C. nutans*) have not examined seed fates [29], [40], [41]. Many introduced plant species may bear elaiosomes, but how elaiosomes influence invader success likely depends on the specific recipient community.

Our results demonstrate that ant foraging can substantially reduce plant recruitment. Ants suppressed recruitment in 10 of 18 of our study species by approximately 40 to 500% (Fig. 3b). Reductions in recruitment of these same exotic plants caused by rodent seed predation translated to lower densities of adult and reproductive age classes for most affected species [15]. Our findings confirm that ants may serve as important filters affecting plant populations in various systems [19], [20], [22], [23]. However, the extent to which local filters influence invasion outcomes depends on whether these filters have differential effects on native versus exotic species’ success. We found that ant seed predation had highly disproportionate effects on recruitment of exotic versus native species, both in terms of the number of affected species and the degree of suppression of each (Fig. 3b). This bias in the seed predation filter appeared to be due to differences in plant traits that largely aligned with plant origins, rather than due to plant origins *per se*, as we found that seed size predicted ant preference independent of plant origins. Seed mass was a strong predictor of ant seed preferences and their impacts on plant recruitment, with ants favoring smaller seed sizes (Fig. 4a). The reason that the filter had greater impacts on exotic versus native plants was that exotics had generally smaller seeds, but why do the exotics have smaller seeds in this system?

We selected our study species to be representative of native and exotic plants common to this system without regard to seed traits. Prior work has shown that ants may select seeds based on nutrient content, surface characteristics, or seed size, with size-dependent selection favoring either larger or smaller seeds depending on ant species and system [42], [43]. Hence, we had no *a priori* reason to control for specific seed characteristics. We found that seed sizes of our nine test species did not differ from those of 16 other exotics found in our system, indicating that our test species were not unusual in this regard. Our exotic test species also differed from the natives in that they exhibited higher emergence rates and earlier peak emergence in the absence of seed predation; traits which were unrelated to seed size. The fact that exotics differed from natives across these traits is suggestive of higher-order filtering effects selecting for certain exotic traits. For example, in some systems exotics have smaller seeds than native species [44], [45], suggesting seed size may be a trait that reflects introduction pathway constraints or possibly regional abiotic or dispersal processes. Collectively, these results suggest that plant traits can help predict invader success within local communities. They also indicate that provenance matters. However, understanding the role of provenance may require integrating across introduction, regional, and local filtering processes [46], and perhaps modifying filter concepts to address invasion-specific factors.

A profound finding from this work is that ant seed predation largely negated recruitment differences between exotics and natives (Fig. 3b), differences potentially advantageous for the exotics. When seed predators were removed, exotics emerged earlier and at higher rates than the natives. However, in the presence of ant seed predation these differences disappeared due to the greater suppressive effect of ants on the exotics. The disproportionate impact of ants on exotic plant recruitment may afford this system greater resistance to invasion. Comparisons of widely disparate grasslands from Germany, California, and Montana found that herbivory and plant competition stifled the advantage of exotics over natives, but when these filters were removed the exotics benefitted far more than the natives [9]. In contrast, rodent seed predators in Montana grasslands had greater impacts on native than exotic plant establishment, suggesting this particular filter favors invaders [16]. These studies suggest that biases in local filters and the degree to which local filters are disrupted across systems may help explain the differential susceptibility of plant communities to invasion [18].

If species origins are unimportant, community filters should act on species independent of provenance, supporting arguments that biological invasions do not differ from native colonization processes [2]. However, there are several reasons to believe that origins should matter. First, the anthropogenic breach in geographic dispersal barriers likely creates a nonrandom introduction filter that favors specific invader traits [47]. Second, even if introduction filters were random, an establishment filter might be expected to favor “weedy” traits such as strong dispersal, rapid growth, or high competitive abilities that facilitate establishment and spread [5]. Finally, biogeographic differences in evolutionary history and ecological context likely result in the introduction of traits that are novel to the recipient range [48]. An extreme example is the introduction of predators to predator-free systems [49], but less extreme examples such as the disassociation of mutualisms like we show for *C. nutans* or the creation of new mutualisms [50] could also result in significant provenance effects. Such novel interactions may be beneficial or maladaptive, but they indicate a substantive role of provenance. Most invasion hypotheses are founded on the notion that introducing organisms into a new range can substantively affect introduction outcomes due to novel interactions [51]. Support for these hypotheses [52] suggests that biogeographic factors are important and species origins do matter [53].

Given the growing evidence that provenance does influence invasion outcomes, a barrier to resolving the debate over whether species’ origins matters appears to be the failure to incorporate species’ origins into community theory. Our results suggest that community assembly theory provides a framework for integrating ecology with invasion biology and transitioning from debates about whether provenance matters to discussions about how provenance matters [54]. Moreover, local filters can identify species traits important for invader success in the context of local community processes. This contrasts with efforts to identify invader traits independent of community context, which have achieved limited success [5], [6]. Nonrandom effects of local filters with regard to species’ origins may indicate a role of higher-order filters like introduction, establishment, or regional abiotic factors, which can condition the traits of exotics entering the local species pool. Modifying community assembly theory to replace the geographic dispersal filter with introduction (which address anthropogenic dispersal constraints) and establishment filters and examining invasions in the context of the full set of filters provides a means for merging biogeography with community assembly theory to advance invasion biology.

## Supporting Information

Figure S1 Data for ant seed preference near nest.
(XLS)

Figure S2 Data for seed fates from basuras.
(XLS)

Figure S3 Data for ant seed removal from Petri dishes.
(XLS)

Figure S4 Data for seed mass versus other variables.
(XLS)

Figure S5 Data for seedling emergence.
(XLS)
