# Correction: Are Local Filters Blind to Provenance? Ant Seed Predation Suppresses Exotic Plants More than Natives

**DOI:** 10.1371/journal.pone.0110725

**Published:** 2014-10-07

**Authors:** The PLOS ONE Staff

Figure 2 is incorrect. The authors have provided a corrected version here.

**Figure 2:**
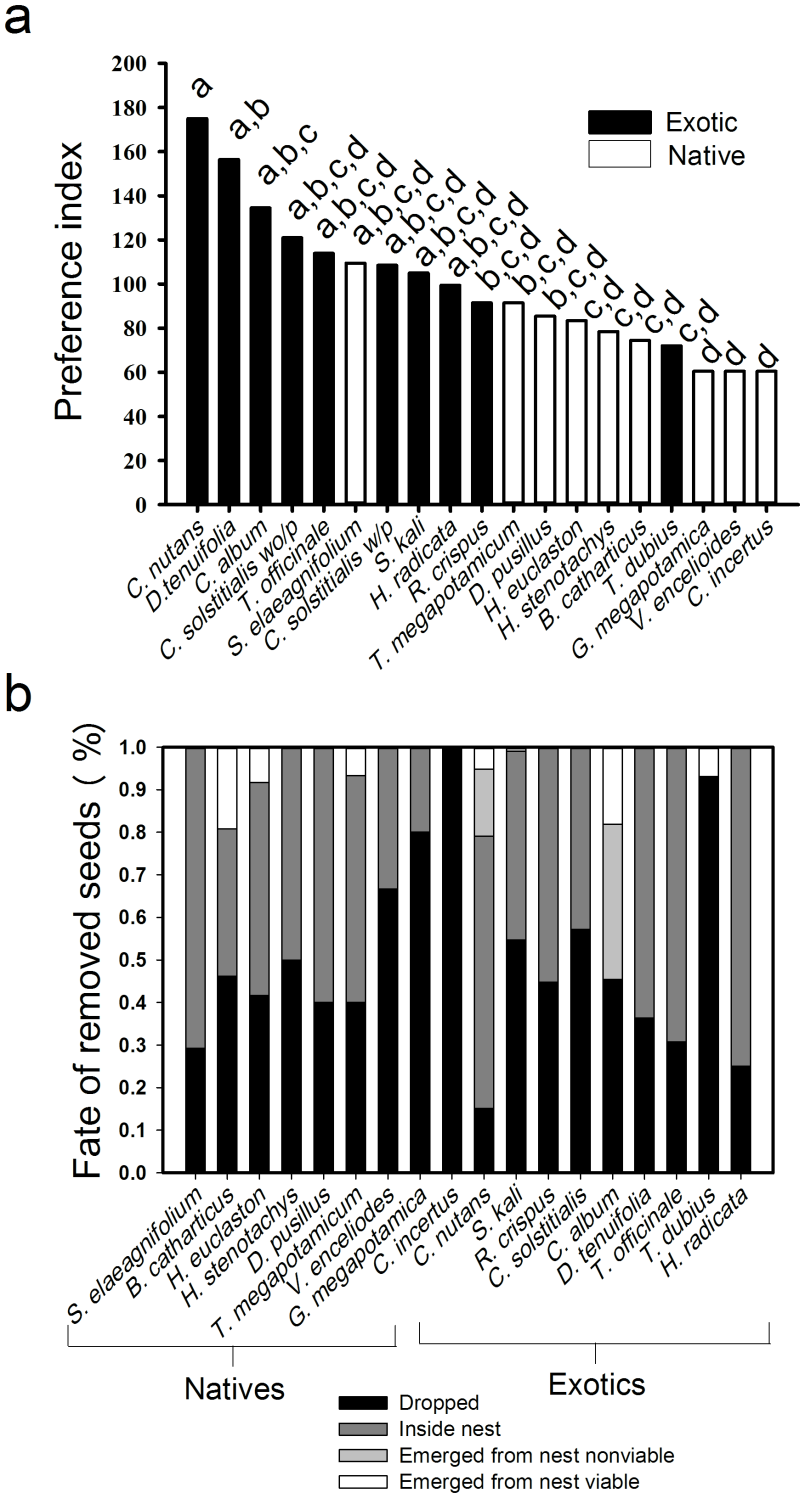
Ant seed preferences and their effects on seed fates. a) Preference of ants for native and exotic seeds based on seed removal from experimental seed depots set out over five days. Species that share letters above bars were not significantly different (Friedman’s test, α  =  0.05). b) Fates of native and exotic seeds removed by ants during 60 min observations of experimental depots placed near nests.
